# Association of Transcriptomic Signatures of Inflammatory Response with Viral Control after Dendritic Cell-Based Therapeutic Vaccination in HIV-1 Infected Individuals

**DOI:** 10.3390/vaccines9070799

**Published:** 2021-07-19

**Authors:** Csaba Fehér, Roque Pastor-lbáñez, Lorna Leal, Montserrat Plana, Mireia Arnedo, Henk-Jan van den Ham, Arno C. Andeweg, Rob A. Gruters, Francisco Díez-Fuertes, José Alcamí, Patrick Aloy, Felipe García

**Affiliations:** 1 Institute for Research in Biomedicine (IRB Barcelona), The Barcelona Institute for Science and Technology, 08028 Barcelona, Spain; paloy@irbbarcelona.org; 2 Infectious Diseases Department, Hospital Clinic, IDIBAPS, University of Barcelona, 08036 Barcelona, Spain; laleal@clinic.cat (L.L.); frandifu@icloud.com (F.D.-F.); ppalcami@isciii.es (J.A.); fgarcia@clinic.cat (F.G.); 3 Retrovirology and Viral Immunopathology Laboratory, AIDS Research Group, IDIBAPS, Hospital Clinic, University of Barcelona, 08036 Barcelona, Spain; rpastor@clinic.cat (R.P.-l.); mplana@clinic.cat (M.P.); marnedo@vhebron.net (M.A.); 4 Department of Viroscience, Erasmus MC, 3000CA Rotterdam, The Netherlands; h.vandenham@enpicom.com (H.-J.v.d.H.); a.andeweg@erasmusmc.nl (A.C.A.); r.gruters@erasmusmc.nl (R.A.G.); 5 National Center for Microbiology, Instituto de Salud Carlos III, 28222 Majadahonda, Spain; 6 Institució Catalana de Recerca i Estudis Avançats, 08010 Barcelona, Spain

**Keywords:** dendritic cell-based therapeutic HIV-1 vaccine, differential gene expression, mRNA, miRNA, gene set enrichment analysis

## Abstract

Systems vaccinology has seldomly been used in therapeutic HIV-1 vaccine research. Our aim was to identify early gene ‘signatures’ that predicted virus load control after analytical therapy interruption (ATI) in participants of a dendritic cell-based HIV-1 vaccine trial (DCV2). mRNA and miRNA were extracted from frozen post-vaccination PBMC samples; gene expression was determined by microarray method. In gene set enrichment analysis, responders showed an up-regulation of 14 gene sets (TNF-alpha/NFkB pathway, inflammatory response, the complement system, Il6 and Il2 JAK-STAT signaling, among others) and a down-regulation of 7 gene sets (such as E2F targets or interferon alpha response). The expression of genes regulated by three (miR-223-3p, miR-1183 and miR-8063) of the 9 differentially expressed miRNAs was significantly down-regulated in responders. The deregulation of certain gene sets related to inflammatory processes seems fundamental for viral control, and certain miRNAs may be important in fine-tuning these processes.

## 1. Introduction

Therapeutic vaccines are among the most promising HIV cure strategies. They aim to boost the immune system of infected individuals to control viral replication without the need for long-term antiretroviral treatment (ART). Although, this goal has not been fully achieved by any of the numerous tested vaccine candidates as yet [1], there have been some partially successful studies published [2].

An important part of the history of most known vaccines is that of trial-and-error, and the development of therapeutic vaccines against HIV-1 is no exception either. Recently, however, novel methods that can complement or even substitute this empirical strategy are gaining popularity. The use of omics technologies in this field gave place to the birth of “systems vaccinology”, an approach that gives good grounds for expecting a significant improvement in the identification of surrogate markers of response and in the vaccine development pipeline [3,4].

Systems vaccinology has proven to be useful in multiple infectious diseases, such as influenza [5], yellow fever [6], hepatitis B [7], or ebola [8]. In one report, a dendritic cell-based (DC) HIV-1 vaccine caused a significant transcriptomic shift in peripheral blood mononuclear cells (PBMC) in HIV-1 infected vaccinees as compared to pre-vaccination state or healthy controls [9]. Another study demonstrated the upregulation of certain genes related to direct cell recognition in NK cells of healthy individuals that had received a modified vaccinia Ankara (MVA) vectored therapeutic HIV-1 vaccine [10]. Recently, changes in gene expression of HIV-1 infected patients who received a DC therapeutic vaccine were shown to be correlated with the post-vaccine peak viral load (VL) [11]. 

Certain microRNAs (miRNA) have been associated with HIV infection [12,13], and disease progression [12,14,15,16,17,18], and some miRNAs were found to be upregulated in HIV-exposed seronegative individuals [19]. However, to the best of our knowledge, there are no published data as yet on miRNA expression in therapeutic HIV-1 vaccine recipients, nor has any miRNA been associated so far with viral control in patients after the withdrawal of ART. Our aim in this study was to analyze mRNA and miRNA expression profiles in participants of a partially successful DC therapeutic HIV-1 vaccine study (DCV2) [2]. We were looking for early gene ‘signatures’ that could be associated with virological response.

## 2. Materials and Methods

### 2.1. Study Subjects and Samples

The present study was performed on data and samples belonging to participants of the DCV2 trial [2]. Thirty-six patients partook in this study, all of them receiving ART, with CD4+ T lymphocyte count > 450 cells/mL and undetectable VL at the beginning of the study. They were randomized 2:1 to receive 3 doses of a vaccine based on peripheral blood monocyte-derived dendritic cells (MD-DC) pulsed with heat-inactivated autologous HIV-1 virions (DC-HIV-1 group) or unpulsed MD-DC (DC-control group) at weeks 0, 2, and 4. In the DC-HIV-1 group, antiretroviral treatment was stopped at week 0 in 12 patients (ATIw0-DC-HIV-1 group) and at week 4 in the other 12 patients (ATIw4-DC-HIV-1 group). These two different schedules were selected to assess whether ART could have any influence on the response to immunizations. Because a significant difference in plasma VL changes or HIV-specific T cell responses between these two schedules were not observed, immunized patients were analyzed in the original study as a single group [2]. One patient in the DC-control group was excluded from the analysis because of consent withdrawal before receiving any immunization. 

Participants were followed for 48 weeks after treatment interruption. Patients with a ≥1 log10 copies/mL drop of VL at week 12 of ATI were defined as “responders”, and patients with a <1 log copies/mL drop of VL were defined as “non-responders”. When VL was not available at week 12 of ATI, the last observation was carried forward for the missing VL (LOCF method).

PBMC were collected from study participants one week before each vaccine dose (that is, on weeks-1, 2, and 3). Monocytes were isolated from these samples and were incubated with GM-CSF, cAMP and IL-4 for 5 days at 37 °C to induce the transformation of cells into MD-DC. These MD-DC were used for fabricating the vaccines. The remaining monocyte-depleted PBMC were frozen at −80 °C. For the present study, samples from week 3—that is, one week before the last vaccine dose—were used. 

### 2.2. mRNA Extraction, Quality Control, and Microarray Experiments

Total RNA was isolated from TRIzol^®^ homogenates of frozen monocyte-depleted PBMC using the RNeasy^®^ kit (Qiagen©, Hilden, Germany), according to the manufacturer’s instruction, as previously described [9]. Briefly, 250 uL of absolute ethanol was added to the aqueous phase after TRIzol^®^ separation and applied to RNeasy spin columns for purification. RNA concentrations and OD 260:280 nm ratios were measured with the NanoDrop^®^ ND-1000 UV–vis spectrophotometer (NanoDropTechnologies, Wilmington, NC, USA). RNA integrity and purity were assessed with an RNA 6000 Nano assay on the Agilent 2100 bio analyzer (Agilent Technologies, Santa Clara, CA, USA). An RNA integrity number > 8.0 was considered acceptable for RNA quality. Total RNA was used as input for the messageAmp labeling kit, and the resulting cRNA was hybridised onto Affymetrix Human Genome U133 Plus 2.0 microarray chips (Affimetrix, Santa Clara, CA, USA) [9].

### 2.3. miRNA Extraction, Quality Control, and Microarray Experiments

miRNA was isolated from frozen monocyte-depleted PBMC using the mirVana™ miRNA Isolation Kit (Invitrogen™, Fisher Scientific, Waltham, MA, USA). The quality control was performed in the 4200 TapeStation system (Agilent, Santa Clara, CA, USA). Samples with high quality were selected for downstream applications (RIN > 7) and conserved at −80 °C. To obtain the miRNA expression profile, we used the Affymetrix GeneChip miRNA 2.0, corresponding to HsMir v1s520779F custom array containing 1738 mature microRNAs and 2333 other small RNAs.

### 2.4. Statistical Analysis

Raw gene expression data was submitted to Robust Multichip Average (RMA) normalization. Data was adjusted by quality metrics [20], batch, scan batch, and sex. Differential gene expression was analyzed with the Bioconductor [21] package “limma” [22] for R (version 3.4.1, R Foundation for Statistical Computing, Vienna, Austria). Statistical significance was defined as a *p*-value adjusted for multiple comparisons < 0.05. Adjustment for multiple comparisons was performed by the Benjamini–Hochberg procedure. 

Gene set enrichment analysis was performed with the software GSEA v3.0 of the Broad Institute, using the molecular signature database MSigDB (v6.0) hallmark gene set (hgs) collection. Pre-ranking of the gene list was performed according to the t-statistics of the differential expression (DE) analysis. Significant differences were defined by a family-wise error rate (FWER) < 0.05. 

In the comparison of miRNA expression, significant DE was defined first identically as in the mRNA analysis (*p*-value adjusted by means of the Benjamini–Hochberg procedure for multiple comparisons < 0.05). A less strict significance criteria was also defined as an unadjusted *p*-value < 0.05 and a log absolute fold change > 0.5. The genes regulated by significantly differentially expressed miRNAs were identified using the “multiMIR” [23] R package of Bioconductor employing three databases with validated miRNA-target interactions: miRecords, miRTarBase and TarBase. 

To contrast the results of mRNA and miRNA data, we performed a GSEA on the pre-ranked gene list used for the original GSEA, using the genes regulated by the DE miRNAs as gene sets. 

## 3. Results 

### 3.1. Patients and Samples

**Figure 1:**
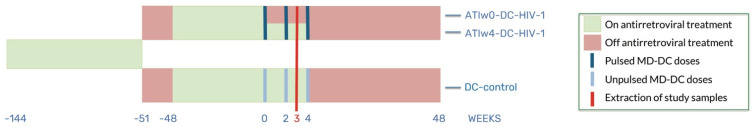
Protocol outline of the DCV2 trial and the time of extraction of the samples for the present study. Patients in the DC-HIV-1 groups received three doses of dendritic cells pulsed with heat-inactivated autologous HIV-1 virions at weeks 0, 2 and 4, and patients in the DC-control group received unpulsed dendritic cells. Half of the patients in the DC-HIV-1 group stopped antiretroviral treatment at receiving the first vaccine dose (ATIw0-DC-HIV-1) while the other half (ATIw4-DC-HIV-1) and the DC-control arm continued treatment until the last vaccination. Samples for the present study were collected at week 3.

Thirty-six chronic HIV-1 infected patients were initially recruited to the DCV2 study. Twenty-four of them received three doses of a vaccine based on peripheral blood monocyte-derived dendritic cells (MD-DC) pulsed with heat-inactivated autologous HIV-1 virions (DC-HIV-1 group), 11 of them received three doses of unpulsed MD-DC (DC-control group), and one patient withdrew consent before receiving any immunization. The main virological end-point was the change in VL at week 12 of ATI with respect to the pre-ART set point (delta set point). Patients with a delta set point ≥ 1 log10 copies/mL were defined as “responders”, and those with a delta set point < 1 log copies/mL were defined as “non-responders”. In this study we analyzed mRNA and miRNA expression profiles of monocyte-depleted peripheral blood mononuclear cells (PBMC) collected after the second vaccine dose. Figure 1 resumes the protocol of the DCV2 trial and indicates the time of sample collection for the present study. 

Of the 35 patients that completed the study, 15 were classified as responders and 20 as non-responders. Of the 15 responders, 12 belonged to the DC-HIV-1 group and 3 to the DC-control group, and of the 20 non-responders, 12 belonged to the DC-HIV-1 group and 8 to the DC-control group. All samples for mRNA analysis showed a good quality. One of the miRNA samples belonging to a subject in the DC-HIV-1 group had to be excluded from the analysis after the quality control and normalization steps. The rest of the miRNA samples showed a good quality.

### 3.2. mRNA Analysis

**Figure 2:**
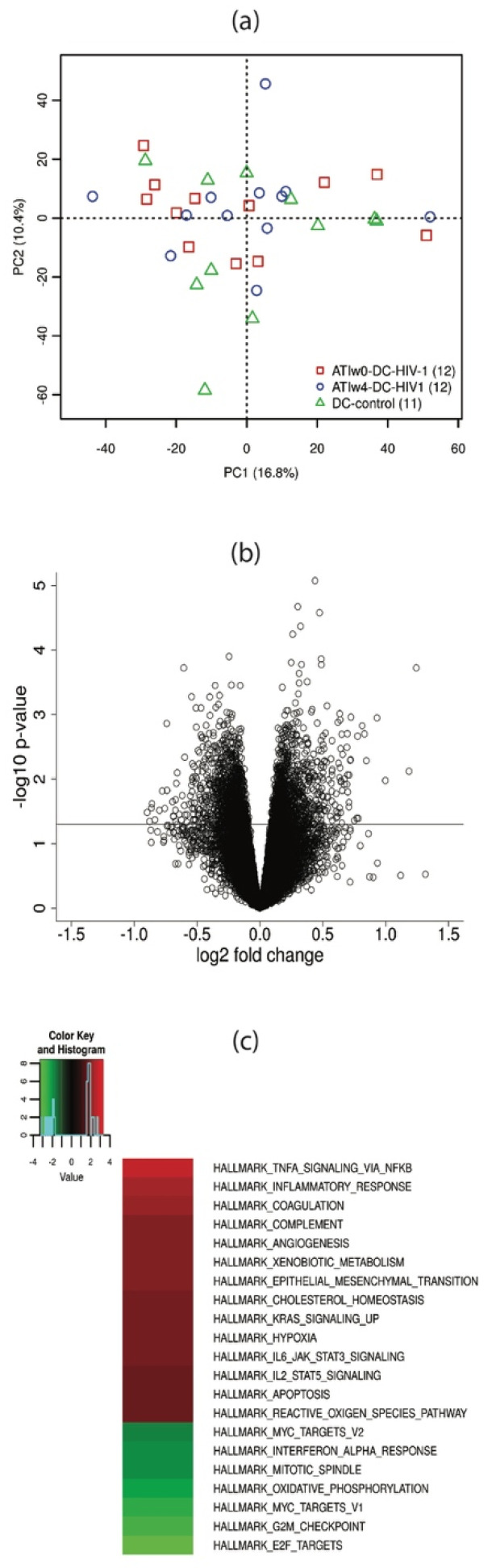
Differential expression analysis of mRNA between responders and non-responders in the DCV2 trial. (**a**) Principal component analysis of gene expression. Colors represent different treatment groups. No clear clustering can be observed according to the main principal components. (**b**) Volcano plot of the comparison of mRNA expression between responders and non-responders. The horizontal line corresponds to an unadjusted *p*-value of 0.05. Differential expression did not achieve statistical significance defined by an adjusted *p*-value > 0.05 in any case. (**c**) GSEA analysis of non-responders vs. responders, using the molecular signature database MSigDB (v6.0) hallmark gene set collection of the Broad Institute. At a significance level defined by a family-wise error rate (FWER) < 0.05, 14 gene sets were up-regulated (red) and 7 were down-regulated (green) in responders as compared to non-responders. Color intensity corresponds to the magnitude of the normalized enrichment score (=actual enrichment score divided by the mean of the enrichment scores against all permutations of the dataset).

mRNA expression was determined for 22,486 genes. We performed a principal component analysis (PCA) of gene expression of the 35 samples, and did not observe clustering of samples according to basic demographic variables or treatment group (Figure 2a). 

We observed no significantly differentially expressed genes (adjusted *p*-value < 0.05) in pairwise comparisons of the treatment groups. The gene set enrichment analysis (GSEA) performed on the treatment groups did not reveal any significantly up- or down-regulated gene sets in vaccinated DC-HIV-1 groups with respect to the control group (data not shown). 

Next, we carried out the comparison of responders and non-responders irrespective of the treatment they received. No DE genes were found between these two groups either (Figure 2b). In the GSEA, however, we identified various Broad hallmark gene sets (hgs) with significant differences between responders and non-responders. Gene sets corresponding to TNF-alpha signaling via the NFkB pathway, inflammatory response, coagulation, the complement system, Il6 and Il2 JAK-STAT signaling, or reactive oxygen-species pathways were up-regulated, while gene sets corresponding to E2F targets, oxidative phosphorylation, or interferon alpha response were down-regulated in responders (Figure 2c).

### 3.3. miRNA Analysis

**Figure 3:**
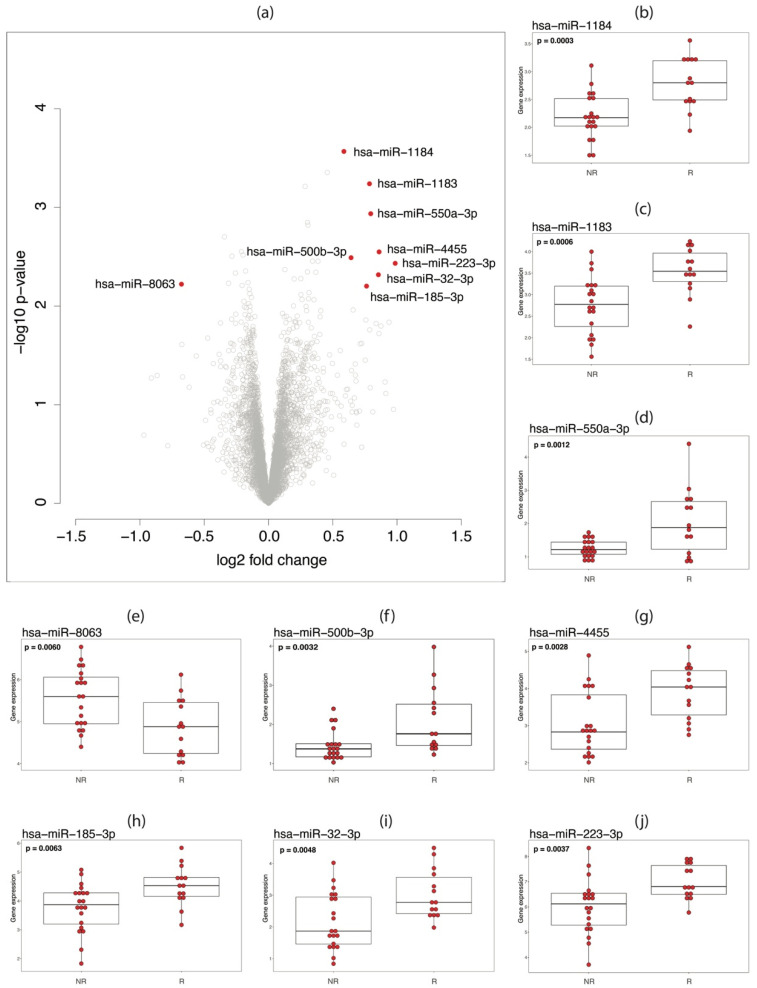
Differential expression analysis of miRNA between responders and non-responders in the DCV2 trial. (**a**) Volcano plot of the comparison of miRNA expression between responders and non-responders. Significant differential expression was defined by an unadjusted *p*-value < 0.05 and a log absolute fold change > 0.5. The nine miRNAs fulfilling these conditions are labeled and marked in red. (**b**–**j**) Expression of the 9 most significantly differentially expressed miRNAs marked in panel *a*. Box plots and individual gene expression values (red dots) are plotted for non-responders (NR) and responders (R) in each case. Unadjusted *p*-values are given in each plot.

In the miRNA expression analysis, no DE miRNAs were observed between responders and non-responders using adjusted *p*-value < 0.05 as significance threshold. Using less strict significance criteria (unadjusted *p*-value < 0.05 and a log absolute fold change > 0.5), nine DE miRNAs were identified between the two groups (Figure 3a). Eight of them (miR-32-3p, miR-185-3p, miR-223-3p, miR-500b-3p, miR-550a-3p, miR-1183, miR-1184, and miR-4455) were overexpressed and one (miR-8063) was underexpressed in responders with respect to non-responders (Figure 3b–j). 

With MultiMIR we identified 899 unique genes that are regulated by one or more of these miRNAs. Table S1 shows the list of validated mRNAs regulated by each of these miRNAs. 

### 3.4. Combined mRNA—miRNA Analysis

**Figure 4:**
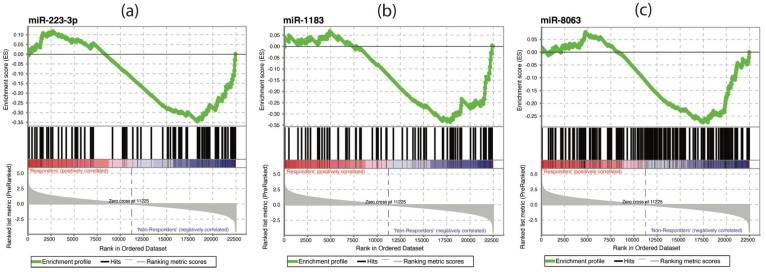
Significant enrichment plots in the GSEA performed with gene sets including genes regulated by the significantly differentially expressed miRNAs. (**a**) The gene set defined by genes regulated by miR-223-3p was significantly down-regulated in responders (*p* = 0.014). (**b**) The gene set defined by genes regulated by miR-1183 was significantly down-regulated in responders (*p* = 0.030). (**c**) The gene set defined by genes regulated by miR-8063 was significantly down-regulated in responders (*p* = 0.024).

Next, we performed a GSEA on the mRNA data with gene sets defined by the genes regulated by the 9 DE miRNAs. We observed that the expression of genes regulated by miR-223-3p, miR-1183 and miR-8063 was significantly down-regulated in responders as compared with non-responders (Figure 4). 

We explored the overlap between the genes regulated by the nine DE miRNAs and the 50 hgs used in the first GSEA analysis. We identified 31 overlapping hgs, and we observed that the miRNA, whose regulated genes showed most overlap with hgs, was miR-223-3p (23 gene sets). Ten out of these 23 hgs (43.5%) were also significantly de-regulated in the GSEA performed with these gene sets—eight of them up- and two of them down-regulated. In addition, three of the four hgs that overlapped with genes regulated by miR-8063 were up-regulated in the GSEA (Table S2).

## 4. Discussion

The transcriptomic regulation of biological processes taking place during HIV infection has been investigated for some time, but there are still many unknown and controversial elements of this rather complex issue. Infection by HIV-1 evokes an immune response from the host that is both qualitatively and quantitatively different from other viral infections, given that HIV-1 targets the very immune cells that would normally be the main actors of infection control. The consequence is a dynamic rhapsody of pro- and anti-inflammatory processes that are rather difficult to disentangle, and the addition of ART, ATI, or immunological interventions to the equation does not make things simpler. 

A previous report showed that immune and inflammatory pathways were down-regulated in PBMC after starting ART in HIV-1 infected patients, as well as gene sets related to cell cycle, apoptosis, mitogenic signaling, or the regulation of the response to hypoxia. [24] Similar results were observed in another study on PBMC where JAK-STAT signaling, oxidative phosphorylation, and apoptosis were observed to be down-regulated after ART initiation [25]. In the case of ATI, however, increased expression of genes related to immune and inflammatory responses was observed in PBMC in yet another study, especially in those patients with a greater decrease in CD4+ lymphocyte count after ATI [26]. 

Therapeutic HIV-1 vaccines also seem to activate inflammatory pathways, even in the absence of a clear clinical benefit, and these changes may even exceed in magnitude those evoked by the virus itself. De Goede et al. showed that a DC therapeutic vaccine caused a major transcriptomic shift in vaccinees by week 5 after starting vaccinations, which was not further modified after ATI—and the consequent viral rebound. [9] Moreover, they showed that most of this shift was due to the activation of inflammatory and immune response pathways that remained basically unchanged after ATI [9]. Another recent report showed that inflammatory pathways were down-regulated during the first few weeks of a vaccination schedule with a similar DC therapeutic vaccine, followed by a significant up-regulation by 12 weeks after vaccination (but before ATI). [11] In this last study, it was also observed that the expression of these inflammatory modules correlated with post-ATI peak VL, suggesting a relation between greater inflammatory response and poorer post-ATI viral control. [11] On the contrary, we observed that the up-regulation of inflammatory and immune gene sets in participants of a DC therapeutic vaccine trial was associated with a better viral control at week 12 of ATI, independently of the study group. Additionally, we did not find significant differences in transcriptomic profiles between the vaccinated and control groups. This latter finding—since our analysis was the first one comparing transcriptomics of two groups of patients receiving vaccination with MD-DC (with or without inactivated HIV-1 virions)—needs to be confirmed in future studies. 

It is accepted that miRNAs regulate protein synthesis on a post-transcriptional level. Traditionally, they are considered to exert a negative effect by repressing translation of their target mRNAs, but there is evidence of miRNA-driven activation of the translation process as well. [27] We observed that three of the nine DE miRNAs in our study regulate the translation of genes that participate in biological processes already significantly deregulated on the transcriptional level. The miRNA-driven post-transcriptional regulation may enhance the effect of the transcriptional regulation in some cases, or counteract it in others. This double mechanism of gene expression regulation indicates the importance of the biological processes affected by these groups of genes. 

miR-223-3p was the DE miRNA, whose regulated genes were most DE between responders and non-responders on a transcriptional level. In addition, the genes whose translation is regulated by this miRNA participate in various processes that were found to be significantly deregulated in the GSEA of the mRNA data. miR-223 is a much-studied miRNA that is involved in many biological processes, such as hematopoiesis, blood cell differentiation and activation, and the fine-tuning of the inflammatory response [28]. Its levels are significantly altered in various diseases, such as diabetes mellitus, rheumatoid arthritis, hematologic and solid organ cancer, chronic liver disease, or infections [29]. However, the exact roles miR-223 plays in each of these scenarios are sometimes unclear. It is fundamentally considered an attenuator of inflammation in general, but depending on the cell type and the situation it may interfere with numerous pathways in different ways. For this reason, differential expression results largely depend on study design.

miR-223 seems to be able to directly target HIV-1 and inhibit the expression of its genes in resting CD4+ cells [30]. Apart from this, different studies reported plasma miR-223 expression in HIV-1 infected individuals to be higher [12], lower [13], or not significantly different [18] when compared to healthy subjects. In PBMC, miR-223 was observed to be under-expressed in the presence of HIV-1 infection [19], while in another study it was over-expressed in HIV-1 infected patients as compared to uninfected controls [17]. Moreover, in the first of these two studies, the amount of miR-223 was found to be overexpressed in HIV-1 infected plasma, which underlines the importance of the type of tissue analyzed [19]. 

Our results suggest that for a better viral control after ATI, an increased but controlled inflammatory response is fundamental. Genes participating in multiple pro-inflammatory processes were up-regulated in responders, at the same time as miRNAs regulating immunological processes were over-expressed in these patients. In turn, these miRNAs regulated the translation of certain groups of genes already deregulated at the transcriptional level, thus modulating the implicated inflammatory pathways.

Our study has some limitations. First, mRNA and miRNA extraction and sequencing were not performed in the same institution, which may have added an extra variability to our data. Second, the PBMC samples used were monocyte-depleted, which may have affected the viability of the cells and the observed expression levels after in vitro culture prior to freezing. Third, due to unavailability of MD-DC samples used for vaccine fabrication, we could not analyze their cytokine and interferon expression profiles, which could have contributed to a better understanding of our findings. Fourth, we only analyzed one time point after two doses of vaccine and could not explore temporal changes in expression patterns. Fifth, by the time of sample collection, ART had already been stopped in half of the patients in the DC-HIV-1 group, and gene expression may have been influenced in this group by the immune response to the incipient viral rebound due to treatment withdrawal. In any case, we observed that the up-regulation of inflammatory and immune gene sets at this time-point was associated with a better viral control after ATI.

## 5. Conclusions 

In conclusion, we observed that the up-regulation of gene sets related to the inflammatory response was associated with better viral control in participants of a partially successful DC therapeutic vaccine trial. miR-223, among other miRNAs, seems to play an important role in optimizing this immune response. 

## Data Availability

mRNA and miRNA expression data are available at www.ebi.ac.uk/arrayexpress/experiments/E-MTAB-10414/ and at www.ebi.ac.uk/arrayexpress/experiments/E-MTAB-10421/, respectively.

## Supplementary Materials

The following are available online at https://www.mdpi.com/article/10.3390/vaccines9070799/s1. Table S1: Genes regulated by the nine significantly DE miRNAs. Table S2: The overlap between the genes regulated by the 9 DE miRNAs and the significant gene sets obtained in the GSEA performed with the Broad hallmark gene sets.
