# Supplementary material for: Association of Transcriptomic Signatures of Inflammatory Response with Viral Control after Dendritic Cell-Based Therapeutic Vaccination in HIV-1 Infected Individuals
Source: Vaccines (Basel). 2021 Jul 19;9(7):799. doi: 10.3390/vaccines9070799 (PMC8310264; doi:10.3390/vaccines9070799)
Supplement: Supplementary file 1 [file vaccines-09-00799-s001.zip › vaccines-1259639-supplementary.pdf]

Table 1. Genes regulated by the nine significantly DE miRNAs.

miRNA	Number of validated regulated genes	Regulated genes
miR_32_3p	118	RL6IP6, ATAD5, AZF1, BAAT, BBS10, BTBD3, C5ORF24, C7ORF60, CAPZA2, CARKD, CC2D2A, CCDC71L, CELF1, CHORDC1, CIDEC, COL23A1, CREBL2, CYB5R4, DYNC1LI1, E2F3, EFCAB14, EID1, FAM169A, FAT3, FBLN2, FGFR1OP, FOXC1, FZD6, GPBP1L1, GPC6, GPSM2, HBP1, HDDC2, HMGN1, HNRNPA3, HNRNPR, HOXA10, HOXD11, IL7R, INIP, KIAA1614, KIAA1958, KIF3A, KIF5B, LAPTM4B, LINC00346, LYN, MAP3K12, MED10, MED12L, MKL2, MON1B, MRPL36, MYADM, N4BP1, NAMPT, NCALD, NCOA7, NHS, PAXBP1, PCDHB16, PEG10, PHC3, PHKA1, PPARGC1A, PPIF, PPIL2, PPP1R15B, PPP6R3, PPWD1, PROSC, PTP4A1, RANGAP1, RBPJ, RDH11, RFX7, RNF125, RORA, RSL24D1, SBNO1, SETBP1, SF3A1, SGMS1, SIGLEC9, SMTN, SNX24, SNX4, SPAST, SRSF10, SUMO2, TCF7L2, TM4SF1, TMEM192, TMEM2, TMEM30A, TMEM67, TMF1, TRPC5, TSR1, TVP23C, TWF1, UBE2S, UHMK1, USMG5, USP37, VLDLR, VPS33A, VPS4A, WDR37, WNK1, WTIP, YWHAE, YWHAH, ZDHHC20, ZNF292, ZNF410, ZNF567, ZNF573
miR_185_3p	67	ABCC5, ADCY9, ADRBK2, AKAP2, ANKRD13B, ARHGAP40, ASB6, BARHL1, CACNA1C, CERS1, CNBP, CNOT3, DDX39B, FAM151B, FARSA, FOLR1, GK5, HIST1H1B, HMGA1, HSP90AB1, HSPA14, IDS, ITPRIPL1, MAPK1, MKNK2, MTHFSD, NF2, PALM2-AKAP2, PEX26, PIAS4, PIM3, POLR3G, POMGNT1, PPP1R11, PPP2CA, PSMB6, PTDSS2, RAB5B, RGS6, SCAF4, SCN2B, SEMA3F, SERF1A, SERF1B, SFT2D2, SLC10A3, SLC27A1, SOX4, STAC, SYNGR2, TET3, TEX261, TFPI, THRA, TIMM8A, TMEM109, TMEM63C, TRIP10, TXNRD3NB, U2SURP, UNK, USP37, VAV3, VPS37C, WBSCR16, ZBTB7A, ZNF689
miR_223_3p	85	ABCB1, ARL8B, ARTN, ATM, BAG2, C9orf40, CACNG8, CAPRIN1, CARM1, CCL3, CDC27, CDK2, CDS1, CFTR, CHMP2B, CHUK, CXCL2, CYB5A, E2F1, ECT2, EPB41L3, F3, FABP7, FAM60A, FBXW7, FOXO1, FOXO3, GPATCH8, HEXIM1, HSP90B1, IGF1R, IL6, IL6ST, IRS1, ITGB1, LATS2, LIF, LMO2, MDM2, MEF2C, MKNK2, MSMO1, MTRF1L, NAMPT, NFIA, NFIX, NMNAT2, NOVA2, NSUN3, PARP1, PAX4, PAX6, PDZD8, PHF19, POLR3G, PRDM1, PTBP2, RANGAP1, RHOB, RIF1, RRAS2, SCARB1, SECISBP2L, SEPT2, SESN3, SLC2A4, SLC7A5, SMARCD1, SNX24, SP1, SP3, SPPL2A, STAT5A, STMN1, TAL1, TMEM64, TMEM67, TOX, TP53, TRPV2, TWF1, WASL, ZBTB18, ZNF365, ZNF460
miR_500b_3p	171	ABCA6, ABL2, ACOT9, ADCY2, ADM, AGRN, AK1, ALG14, AMMECR1L, ANO6, AP3B2, APOB, ARHGAP27, ARL17B, ARMC12, ARRDC2, ASCC1, BARD1, BTLA, C16orf45, C17orf104, C3orf18, C8A, CCDC134, CCDC142, CCDC80, CCR6, CDCA4, CDK6, CERS4, CHDH, CHMP3, CLIC5, CLMN, COX19, CXorf38, CYTH2, DDX19B, DYNLL2, EFTUD2, ERC1, ESF1, FADS6, FAM213A, FAM229B, FBXL2, FGF1, FLG2, FXN, GEMIN6, GOSR1, GREM2, HDGF, HEBP2, HIF1AN, HLA-E, HNRNPK, ICOSLG, IDS, IGSF9B, IL1RL2, ISCA2, ITPA, ITPKC, JPH2, KAT7, KBTBD6, KCTD15, KDELC2, KDELR1, KIAA0930, KIAA1549, KIAA1551, KIAA1919, LEAP2, MASTL, MED18, METTL2B, MIDN, MOGAT1, MRPL45, MRPL52, MSANTD4, MTMR10, MUC20, MVB12B, MYLK3, N4BP2L2, NIPAL1, NLN, NOVA2, NPEPPS, NUDT3, OPRD1, OPTN, PAICS, PCSK2, PCSK9, PGBD4, PGBD5, PHC3, PIGG, PLEKHA3, POLM, POLQ, POLR3A, POM121L7, PRIM1, PTCH1, PTPLAD2, PXMP4, PYCRL, PYGO1, QPRT, RANGAP1, RBM27, RBM43, REL, RHBDL2, RHOH, RNF103-CHMP3, RNF115, RRAD, RTTN, SAMD8, SAR1A, SENP3, SF3A3, SGOL1, SLC16A10, SLC1A5, SLC26A2, SLC2A6, SLC31A1, SLC35B4, SMOC1, STAR, STAT3, STRN3, TARS, TDRD1, TIGD6, TLDC1 TMEM119, TMEM120B, TMEM55A, TMEM74B, TMOD2, TNFRSF13C, TNFSF8, TNRC6B, TRIM58, UBE2G2, UNC119B, WDR81, WRN, WT1, ZBTB7A, ZBTB8A, ZC3H12C, ZDHHC8, ZFP14, ZFPM1, ZNF439, ZNF460, ZNF491, ZNF554, ZNF74, ZNF786, ZNF845, ZYG11B
miR_550a_3p	40	ABCF2, ACTR1A, C2orf72, CMIP, CNIH, CXCL10, DNAJA3, EFHB, EID2, FEM1B, GTF2E1, HPS4, HSP90AA1, KCNE4, KDELR2, MDM2, MTAP, OR7D2, PKIA, POLR2F, POLR3K, PXMP4, RBM8A, SC5D, SLC11A2, SLC35F6, SYNCRIP, TM4SF19, TMEM50B, TRAF1, TUBD1, TXLNG, URM1, YOD1, YWHAE, YY2, ZC3H12C, ZNF525, ZNF621, ZNF703
miR_1183	77	ALG13, AMACR, ARF6, ARPP19, ATP6V0A2, ATXN7L1, BICD2, C11ORF58, C19orf35, C21orf91, CEP135, CHML, CREB1, CRLF3, CSNK2A1, DCAF7, DEK, DONSON, ELL2, ERBB2IP, FAM53C, FEM1C, FLVCR1, GATA6, GPATCH8, GRAP2, GRHL1, HECTD1, HHLA1, HMGB1, HSPA4L, IL20RB, IPMK, IST1, KIAA0232, KLHDC10, LAMTOR1, LRCH3, MAP3K9, MAPK14, MEOX2, MOB1B, N4BP2, NFATC2, NPTN, NUP188, OSMR, PDPK1, PEX3, PIAS1, PLEKHA1, PLEKHF2, POLR3F, PRAMEF1, PSME4, RACGAP1, RBBP4, RLN1, RWDD2A, SEP15, SERPINA4, SGIP1, SHCBP1, STAT1, STRBP, SYNM, TMED7, TMEM164, TNFSF15, TNKS, TYRP1, UBL3, ZC3H12C, ZC3H6, ZHX3, ZNF35, ZNF608

miR_1184	86	ARL5C, ATG12, BDP1, CAPN1, CEP89, CREBRF, CRK, CRKL, CSNK2A1, DCC, DICER1, DNASE1L3, DRAXIN, EHD3, EIF1AX, ENPP2, ERN1, FAM98A, FBXL20, FEM1A, G3BP1, GCC1, GGA2, GLUL, GPCPD1, GSTO2, HIST1H2BK, HMGA1, HOXA13, IBA57, IDH3A, IL6R, JPH2, KDELR1, KIAA1549, LAX1, LSM14A, LY6G6E, MIS18A, MIS18BP1, MPDU1, MRS2, MTA1, NLRC5, NOA1, NPY4R, NR6A1, NUMB, OTOF, PANK3, PDPR, PHF12, PICALM, PIKFYVE, PLIN5, PLXND1, POLDIP2, RAB1A, RNF11, SESN2, SETD1B, SF1, SLC25A45, SLC30A3, SLC6A4, SMARCE1, SNX9, SRSF9, STX16, SYNRG, TECPR1, TMED10, TMEM109, TMEM132C, TMEM170A, TNFAIP1, UBE2G1, UBE2H, VGLL4, VPS8, XPO5, ZBTB24, ZDHHC6, ZMIZ2, ZNF442, ZNF483
miR_4455	133	ACP1, ADRBK2, ADRM1, AGPAT5, ANO8, APOL6, ARSK, BDH1, BUB1, C16ORF72, C19orf52, CACUL1, CAMK2N1, CAMK4, CASZ1, CBX1, CD40LG, CDH12, CDK15, CDKN1A, CDON, CLEC2D, CNTF, COX6B1, CPM, CUX2, CYP7B1, DBT, DHODH, DIABLO, DMD, DNAJC6, DOCK1, EDA2R, EEA1, EEF2, EN2, FAM117B, FAM69C, FAM83C, FAXC, FGF14, GLI2, GPBP1L1, GXYLT2, IL2RB, JAKMIP2, KANSL1L, KATNAL1, KCNQ3, KCNQ5, KIAA1549L, KIF1C, LEPREL1, LHFP, LIPC, LPP, LRRC10, MAN1A2, MAPK10, MARCH3, MARCH4, MED28, MOCS3, MPEG1, MTHFD1, MTRNR2L11, NACC2, NCAN, NCOA3, NMNAT2, NR2E1, NUP93, ONE-CUT3, PARP2, PAX5, PGBD4, PLAC8, PLEKHA1, PORCN, PPP1R16B, PRIM1, PRKCB, PRRT2, PTCHD1, PYGO1, RAB3C, RAP2B, ROCK2, SAMD9L, SBK1, SEPT6, SESN2, SETD1B, SETD5, SF3B3, SH3TC2, SIM1, SLC16A13, SLC35F6, SLC43A3, SLCO4C1, SPPL3, SPRY4, SYT2, TBX4, TCTE1, TGOLN2, TMED4, TMPRSS6, TNR, TNS4, TRAF3, TTLL7, TXNL4B, TYRO3, UBE2Q1, UBN2, UGT2B4, VAMP4, VANGL1, WNT4, XKR6, YOD1, ZBED3, ZC3H4, ZDHHC20, ZEB1, ZNF131, ZNF382, ZNF529, ZNF556, ZNF878
miR_8063	181	AAK1, ACTB, ACVR1C, ADCYAP1R1, AGO3, ALAD, ALKBH5, AMOTL2, AP5M1, ARHGAP12, ARL5B, ASH2L, ATG9A, ATP6V1C1, AVPR1A, BCL2L11, BDP1, BRPF3, BTF3L4, BTG3, C12orf29, C12orf5, C16orf52, C16orf72, C17orf105, C19orf44, C1GALT1, CALCR, CAPN7, CCDC108, CCDC71L, CCND2, CCNL2, CDC42BPA, CENPH, CENPL, CEP85L, CLSPN, CMTM6, CPT1B, CRNKL1, CTDSPL, CXCL5, CYBRD1, DCTN4, DCTN6, DEPDC1, DEPTOR, DIP2A, DNAJC10, DNAJC21, DST, E2F2, EDIL3, EDN3, EHD4, EIF1AX, ELAVL1, ELAVL2, EMC3, EPHA2, ERBB3, EXOC5, EXOC8, FAM103A1, FAM129A, FAM216B, FAR2, FBXO28, FEM1B, FKBP15, FRK, FSIP2, FUT11, GABRB2, GALM, GATA6, GATAD1, GLRX2, GNG12, GPR151, GRAMD3, HAS2, HEPHL1, HIST1H2BD, HIST1H3E, HNRNPC, HNRNPDL, IFNB1, IL5, IRF2BP2, JAG1, JAZF1, KDSR, KIAA0101, KIAA1614, KIT, L3MBTL2, LIMD1, LMNB2, LRRC4, LSM3, MAP3K2, MAPT, MMS22L, MOCS2, MON1B, MRPS10, MUT, MVK, MYLK, NAA38, NECAB1, NHLRC2, NOX5, NUDT21, ODF4, OTUD7B, PAFAH1B2, PAIP1, PAK2, PAK3, PDE1A, PEG10, PITX3, POLR1A, POU5F1B, PPIL2, PRDM5, PRDX3, PRKAA1, PTPDC1, QRSL1, RCAN1, RNF165, RPS4Y1, SCOC, SEC22C, SEPT2, SETD5, SGCD, SLC19A3, SLC35E2, SLC38A9, SMU1, SPATA13, SPOP, SPRED1, SPRED3, STRBP, SZRD1, TAB2, TACC1, TCF7L2, TENM4, TES, TESPA1, TGFBR1, TIGD2, TMED7, TMEM119, TMEM59, TMEM67, TNFSF15, TRAFD1, TRAPPC4, TSPAN12, TTC39C, TWISTNB, U2AF2, USP48, VIM, WAPAL, WNK1, XKR4, YIPF6, ZDHHC21, ZDHHC23, ZNF426, ZNF573, ZNF770

Table 2. The overlap between the genes regulated by the 9 DE miRNAs and the significant gene sets obtained in the GSEA performed with the Broad hallmark gene sets.

miRNA	Number of regulated genes [n]	Number of Broad hallmark gene sets with significant (FDR q-value < 0.05) gene overlap [n (%)]	Number of overlapping Broad hallmark gene sets that are significantly up- or down-regulated in the mRNA analysis [n(%)]	Overlapping Broad hallmark gene sets up- or down-regulated in the mRNA analysis
miR_32_3p	118	2 (4.0%)	2 (100.0%)	Hypoxia, Myc targets v1
miR_185_3p	67	1 (2.0%)	0 (0.0%)	-
miR_223_3p	85	23 (46.0%)	10 (43.5%)	TNFA signaling via NFkB, KRAS signaling up, G2M checkpoint, Hypoxia, Il2-STAT5 signaling, Inflammatory response, Apoptosis, Mitotic spindle, Epithelial mesenchymal transition, Il6-JAK-STAT3 signaling
miR_500b_3p	171	0 (0.0%)	0 (0.0%)	-
miR_550a_3p	40	0 (0.0%)	0 (0.0%)	-
miR_1183	77	0 (0.0%)	0 (0.0%)	-
miR_1184	86	0 (0.0%)	0 (0.0%)	-
miR 4455	133	2 (4.0%)	0 (0.0%)	-
miR_8063	181	4 (8.0%)	3 (75.0%)	Apoptosis, Cholesterol homeostasis, Epithelial mesenchymal transition
All 9 miRNAs	899	31 (62.0%)	12 (38.7%)	G2M checkpoint, Il2-STAT5 signaling, TNFA signaling via NFkB, Inflammatory response, Myc targets v1, Mitotic spindle, Hypoxia, E2F targets, KRAS signaling up, Apoptosis, Il6-JAK-STAT3 signaling, Cholesterol homeostasis
